# Stakeholder perceptions and operational barriers in the training and distribution of take-home naloxone within prisons in England

**DOI:** 10.1186/s12954-016-0094-1

**Published:** 2016-02-03

**Authors:** Arun Sondhi, George Ryan, Ed Day

**Affiliations:** Therapeutic Solutions (Addictions) Communications House, 26 York Street, London, W1U 6PZ UK; Public Health England, 2nd Floor Skipton House London Road Elephant & Castle, London, SE1 6LH UK; Addiction Psychiatry, Addictions Department, National Addiction Centre, Addiction Sciences Building, 4 Windsor Walk, Denmark Hill, London, SE5 8AF UK

**Keywords:** Naloxone, Opiate-related overdose, Prison

## Abstract

**Background:**

The aim of the study was to assess potential barriers and challenges to the implementation of take-home naloxone (THN) across ten prisons in one region of England.

**Methods:**

Qualitative interviews deploying a grounded theory approach were utilised over a 12- to 18-month period that included an on-going structured dialogue with strategic and operational prison staff from the ten prisons and other key stakeholders (*n* = 17). Prisoner perceptions were addressed through four purposive focus groups belonging to different establishments (*n* = 26). Document analysis also included report minutes and access to management information and local performance reports. The data were thematically interpreted using visual mapping techniques.

**Results:**

The distribution and implementation of THN in a prison setting was characterised by significant barriers and challenges. As a result, four main themes were identified: a wide range of negative and confused perceptions of THN amongst prison staff and prisoners; inherent difficulties with the identification and engagement of eligible prisoners; the need to focus on individual prison processes to enhance the effective distribution of THN; and the need for senior prison staff engagement.

**Conclusions:**

The distribution of THN within a custodial setting requires consideration of a number of important factors which are discussed.

## Background

Naloxone is an opioid antagonist that is used to counteract an overdose of an opioid drug such as heroin. The drug is used as part of an emergency overdose response, and there is evidence that mortality rates can be reduced [1]. The use of take-home naloxone (THN) is seen as part of a package of interventions aimed at identifying and responding to an overdose, including use of rescue breathing and calling medical emergency services. The perceived advantage of THN is that it can be administered by non-medical individuals who have received at least some training. Naloxone can also be seen as a useful medication for illicit drug users as it has no clear potential for abuse. The drug can be administered via intramuscular, intravenous, subcutaneous, or intranasal routes.

The importance of THN within a prison setting is based on the consistent strong links between substance misuse and mortality within relatively short time periods from the point of release from prison. The evidence base has consistently highlighted the strong links between substance misuse and mortality at the point of release. A recent paper [2] suggested that for men released in Sweden, the estimated probability of death within 5 years of release was 10.2 % for substance misusers compared to 3.2 % of non-substance misusers. Similar findings were shown for women, with 6.5 % probability of death for female substance misusers within 5 years of release compared to 2.6 % for non-substance misusers.

Other research has shown that male prisoners are 29 times more likely and female prisoners 69 times more likely to die than the general population, with the first 2 weeks a key period for drug-related mortality [3, 4]. Despite the importance of the first 2 weeks as a key period for intervention, Chang et al. [2] suggest that the strong probability of death relating to alcohol or drugs persists beyond the initial release period for up to 5 years. The authors also suggest that the prevalence rates are not predicted by higher rates of mental health problems, although there is a recognition that being diagnosed with a substance use disorder may ‘mask’ other prevalent mental health issues. A similar study [5] found that following the introduction of opiate substitution therapy (OST) across all prisons in Scotland there was a 40 % reduction in the level of drug-related deaths 12 weeks post-release.

Opiates are involved in 95 % of cases of drug-related death reported to coroners, with relative youth (being aged less than 30 years), white ethnicity, involvement in acquisitive crime (robbery, theft, fraud, burglary, forgery, handling stolen goods), and prison sentences of less than 2 weeks identified as key risk factors [6]. The length of sentence may be a key factor in predicting opioid overdose as very short sentence lengths are unlikely to provide sufficient time for opiate dependence, or other problematic drug use, to be adequately treated once in prison (due to the limited likelihood of accessing OST or any clinical or psychosocial treatment). Moreover, until recently in the UK, short-term sentences of less than 1 year did not require an offender to have a probation supervision order once released from prison, therefore reducing the probability of an intervention being offered at the immediate post-release point.

The importance of an intervention for prisoners at the point of release, and in particular the potential of THN, has been identified as a key harm reduction measure [7]. This study presents the findings from a qualitative research project that examined the implementation of THN in ten prisons in a region of England, as part of a wider resettlement or ‘Through-The-Gate’ initiative. This central government policy, which forms part of the ‘Transforming Rehabilitation’ programme, aims to provide a series of tailored interventions for substance misusers on their arrival into an establishment and throughout their time in custody, as well as providing continuity-of-care arrangements at the point of release in order to support the goals of abstinence and sustained recovery. For known opiate-using offenders, ensuring the availability and provision of THN formed a key component of the programme. The use of THN as part of the ‘Through-the-Gate’ initiative is seen as part of a package of interventions aimed at identifying and responding to an overdose, including teaching prisoners in the use of rescue breathing and calling for medical emergency services.

### Effectiveness of training

The literature on THN largely focuses on community provision, with only a small number of localised studies focusing on its distribution within custodial settings. Published research within the last 10 years includes of a range of small-scale studies and one UK-based randomised-controlled trial (N-ALIVE), whose status was unclear at the time of writing. Commentators have highlighted the paucity of research on THN [7, 8]. Whilst the literature has focused on service user perspectives in the community, there are considerably fewer studies examining service provision amongst prisoners, despite the clear advantages of targeting this group. The focus has been on testing whether the knowledge imparted during a THN training session has been maintained at subsequent points in time thereafter (usually short term from 3–6 months later). Overwhelmingly, this suggests that service users (usually opiate injectors) are better informed and more knowledgeable about the risks of overdose, following the THN training sessions [8–14]. Although a key study examining mortality rates across Massachusetts between 2002 and 2009 found significant reductions in opiate-related deaths following a THN training and education programme [15], a systematic review of the effectiveness of naloxone distribution programmes in the community [10] was unable to comment on the effectiveness of THN as a means of reducing fatal and non-fatal overdose due to methodological weaknesses in the studies included. Overall, the majority of injectors who were trained were motivated to engage with training and were willing to be involved in the management of overdose, either by themselves or with peers. In a sample of 70 patients in Birmingham and London, 80 % of the respondents still had their kits at the 6-month follow-up and reported a high level of knowledge on the risk of overdose [12]. In addition, the majority have been found to be comfortable using THN following the training [16] and are willing to administer the drug when needed [17]. Overall, the THN training has been described as enhancing the empowerment and confidence of drug misusers [9] with the potential to extend the training to their families and carers [11, 18] as the next step in distributing kits to those who may use and need them.

The training curricula are poorly described in the research literature, and there is a concern that studies which show an immediate improvement in knowledge following a training session may have little relevance to an overdose situation sometime in the future. However, previous studies show that training does provide sufficient knowledge for individuals to effectively administer naloxone [10]. Moreover, the training of THN may be reinforcing other life-saving measures such as rescue breathing and awareness of the recovery position, and so bundling the THN training as part of a wider response strategy to overdose may be just as useful.

The aims of this study were fourfold: firstly, to understand how practitioners could best disseminate naloxone kits to prisoners at the point of release back into the community; secondly, to identify barriers in taking up training, including acceptance of kits by prisoners; thirdly, to discuss stakeholder perceptions on the use of THN and finally, to map processes within prisons to identify the most efficient means by which naloxone kits can reach a prisoner at the point of release. It should be noted that the distribution of THN was aimed at potential future drug use once released and back into the community, and there was no expectation that prisoners could access THN kits within the prison itself for use in the establishment. During the time of the study, there had been one possible opioid-related death although this had not been confirmed in the coroner’s report.

## Methods

The analytical approach for this study utilised grounded theory [19–22] to understand the stakeholder voices and perceptions underpinning the THN training and subsequent implementation. The goal was to generate theories to explain why there may be barriers in the delivery of THN within a custodial environment. Grounded theory has a number of key features [23] that allow for a concurrent data collection and analysis process across a potentially wide range of sources. Integral to this approach is the concept of ‘theoretical sensitivity’ which is dependent on the creation of an analytical coding structure that is not based upon ideas or theoretical frameworks prior to the research. Theoretical sensitivity is developed through immersion in the data collected which allows for a deeper and richer understanding of what participants consider significant or important. The analysis is undertaken in ‘real time’, which then influences the direction of the next stage of interviews and is explicitly aimed at the development of a theory through ‘theoretical sampling’ [19]. For this study, the process incorporated a number of formal and structured real-time discussions alongside more informal conversations over a period of 12–18 months, with a range of strategic and operational stakeholders—a mix of healthcare leads and substance misuse service staff—tasked with the implementation of THN across the ten prisons (*n*=17). This allowed for the analysis for ‘constant comparisons’ across the various datasets to allow for similarities or discrepancies in order to develop the theoretical frameworks [19].

A description of the participating prisons is shown in Table 1.

**Table 1:** Description of the prisons

Prison	Security level	Male/female	Modal age group	Modal sentence length	Percentage opiate users of those known to substance misuse treatment services (%)
1	Category D/‘open’	Male	30–39	4–9 years	19
2	Category C trainer	Male	21–29	4–9 years	74
3	Category B/‘local’ and category A offenders	Male	21–29	Un-sentenced/remand	82
4	Category B/‘local’	Male	21–29	Un-sentenced/remand	43
5	Category B/‘local’	Male	21–29	Un-sentenced/remand	63
6	Category C trainer	Male	21–29	4–9 years	39
7	Category B/‘local’	Male	21–29	Un-sentenced/remand	64
8	Category D/‘open’	Male	21–29	4–9 years	16
9	Category B/‘local’ and young offenders (aged under 21 years)	Female	30–39	2–3 years	64
10	Category C trainer	Male	30–39	2–3 years	3

The range of interviewees included prison representatives from healthcare and substance misuse services, alongside strategic leads for NHS England and representatives from Martindale Pharma, who supplied the naloxone injection kits. Four focus groups (*n*=26) of male prisoners were interviewed on their views on participating in the training within 1 month of the first session. Interviews were manually recorded using paper-and-pen methods by one of the research team. The qualitative interviews were facilitated by a local Naloxone Action Group, which was formed to monitor and present progress in implementation, as well as highlighting and addressing any barriers. The membership of the group included prison representatives across substance misuse teams and healthcare services. The group met on a quarterly basis (five meetings in total), and in-between a spreadsheet that included a specific section examining barriers and potential solutions was sent to all prisons, highlighting the number of prisoners trained to use THN. The main issues arising from the spreadsheet were analysed on a thematic basis. The discussions took place at operational and strategic review meetings that specifically addressed a range of implementation issues. These discussions were in part framed by the grounded theory approach which included the need to identify operational aspects from a provider perspective; the routine identification of service user perspectives, including reasons for refusing to be involved in the implementation of THN; and wider perspectives from prison and community-based staff involved with the delivery of THN either in prison or on-release. The study included a range of complementary sources that could form part of a wider narrative [24], including written papers prepared for stakeholder meetings, and performance reporting on the number of prisoners who had been trained prior to release. These papers often formed discussion points within the meetings that allowed for a greater exploration of issues and potential solutions.

Information was collated at each discussion point or meeting with the aim of deriving themes from the data. As part of the grounded theory approach, a broad inductive or data-driven approach was deployed that did not adhere to any pre-conceived themes. The main approach relied on visual node-link mapping to identify themes from the notes taken at meetings and during the formal interview process, as this method has been shown to help gain a better understanding of complex and ambiguous problems by illustrating influence or causality between factors [25, 26]. This approach utilised the key stages advocated in the qualitative research literature [27] through familiarisation with the information collected by immersion in the available data (e.g. through reading interview notes and transcripts) and recording emerging thoughts and recurrent themes; identification of a thematic framework; indexing codes that link to the themes identified; rearranging data according to the part of the themes identified and mapping and interpreting the data by using visual cues to define any emerging issues.

## Results and discussion

This section provides an overview of the key themes emerging from the action research and the ‘real-time’ discussions with stakeholders, including operational/strategic staff and from focus groups of prisoners. A number of key challenges and operational barriers were identified in this process.

### Negative and confused perceptions of THN amongst prison staff and prisoners

Some prisoners interviewed in the focus groups expressed a degree of uncertainty about the concept of THN and what it aimed to achieve. Some interviewees confused naloxone with naltrexone, an opioid antagonist that is used to help people who have stopped drinking alcohol or using opioid drugs to maintain abstinence. More significantly, there was confusion amongst prisoners about the overarching message underpinning the use of THN within a prison context, because the primary goal of many prisoners in a treatment setting was to abstain from using any substance. For instance:

Since I’ve come to this jail, everyone [has been] banging on about getting myself sorted and off the drugs and everything you know. It’s taken a while and I’m in the right place to move forward for once, no more gear – nothing. I’m done with that life….. I’m clean now with no intention of using, so why do I need this? [Male prisoner, Prison 5]

This finding supports the conclusions of community-based research amongst the treatment population [12] that suggests that once abstinence is achieved, any acknowledgement that future drug use is a possibility represents a sign of lack of commitment to recovery. This issue was not specifically covered in the training although did form part of a subsequent ‘question and answer’ response for later sessions. Prisoners also highlighted side effects and the possible unintended consequences of being in the possession of a THN kit once released. Some were concerned about the perceived side effects of using THN, including being ‘…put into an instant rattle’, similar to the ‘dope-sickness’ identified by the wider literature [13, 28]. In addition, prisoners were concerned about the possible criminal-justice consequences of having been found with a THN kit in their possession. This included the fear of police or criminal justice services, such as the Probation Service or the privatised Community Rehabilitation Companies, who may surmise that carrying a kit implied active drug-using behaviour (and therefore breach of the conditions on any supervision arrangement). This was a key concern that influenced prisoners’ perceptions on the need for THN training whilst in prison.

What’ll happen is that I will leave here and as soon I get my feet on the ground the police will stop me, as always, and say…..’What’s this? You must be using again - you’re nicked [arrested]'. [Male prisoner, Prison 2]

Similar perceptions existed amongst some prison-based staff across most disciplines, including healthcare and substance misuse services. Non-health-related prison staff reported some degree of negativity about the implementation of THN. Firstly, there was a perceived disconnect between the stated aim of THN as a harm reduction measure to reduce drug-related deaths and the perception that naloxone could act as a potential incentive or encouragement for using drugs again once prisoners went back into the community. This perception was linked to a ‘safety valve’ hypothesis [29] identified amongst community-based participants suggesting that the lack of potential negative consequences could encourage the misuse of opiates.

Many of my staff understand what we are trying to do here which is get prisoners off drugs and abstinent by the time they leave here…if not abstinent entirely then at least in a positon to consider an abstinent life. For many staff, and it’s not just uniformed staff [prison officers], just handing out Naloxone give out the wrong message that says it’s alright now you can keep on using. I don’t agree with this view myself but I know they [staff] think it. [Healthcare Manager, Naloxone Action Group]

This view was made up of two components: confusion over the abstinence-harm reduction dichotomy described above and practical concerns regarding the equipment (such as the needle) that could be utilised for an illicit drug use. Therefore, there may be a scope to develop a strategy to inform both practitioners and clients of the potential benefits of THN, which includes discussion of how this harm reduction measure is placed within a recovery context.

### Difficulties with the identification and engagement of prisoners to receive THN training

Staff highlighted pragmatic concerns regarding the identification of prisoners with a history of opiate use and the on-going engagement of prisoners across all establishments. The ability to identify eligible prisoners to receive training should be a key component in driving a programme of training. The point at which prisoners received THN training was often driven by pragmatic factors such as finding sufficient prisoners to train, which often worked against the original aim to deliver training to prisoners near to or at the point of release. Some staff tried introducing THN to prisoners on arrival as part of the induction process. This was seen as relatively ineffective as induction was not conducive to the introduction of THN in many prisons, because there was limited opportunity to explain the rationale or to discuss prisoner concerns in a confidential manner. In addition, staff suggested that prisoners were overwhelmed with the number of priorities competing for their attention at the point of induction, and this led to the fear that key messages regarding THN would be ‘lost’ if it was introduced too early. However, the staff interviewed preferred to introduce THN either during the assessment or as part of the care/recovery-plan process, where there was a greater opportunity to directly address concerns and misunderstandings. The staff had considered creating bespoke node-link maps aimed at addressing the barriers to engagement and encouragement of training. This evidence-based visual approach to presenting and discussing information already forms part of the tailored, therapeutic package of psychosocial interventions offered to prisoners as part of the UK Routes to Recovery initiative [30].

Stakeholders also highlighted difficulties in identifying opiate users in order to target them for training. Systems were not often established that allowed an automatic ‘flag’ to be added to prisoners known to have a history of opiate use. There was neither a routine nor a systematic way to track prisoners’ activities from their arrival to discharge that would include information on training and other needs. All prisons created simple, bespoke spreadsheets of information that were held by one staff member who was involved in the project, and concerns were raised about the need to place the initiative on a more sustainable footing. One prison was able to identify prisoners 8 weeks before release and start of the training programme and provided supplementary ‘clinics’ just before release in order to test and update knowledge if required. Here, prison staff undertook additional focus groups and one-to-one activity with prisoners to reinforce the messages derived from the training. The prison staff reported that prisoners who engaged with substance misuse services were more likely to be contacted than those who were disengaged or had completed their treatment and were discharged from the service. Moreover, attempts at engaging other cohorts of prisoner (for example, through prison-wide leaflet drops) failed, as few prisoners responded positively to wider prison-based initiatives. In one establishment, the prison tested a leaflet drop amongst all prisoners and found only eight prisoners out of a total of 540 leafleted expressed an interest in the THN training, and of these, only four attended the subsequent training session.

The staff also discussed targeting specific sub-groups of prisoners (for example, at different stages in the prison sentence) and the difficulties inherent in such an approach. Not only does this approach rely on the intelligent use of management information systems (as discussed above) but also on the staff, who are required to establish de facto criteria as to who should receive the training. For example, stakeholder interviews suggested that the maximum impact of THN training would be in prisoners who are about to be released. As THN packs could not be transferred across establishments, prisoners who moved to other prisons before release were therefore deemed ineligible for training, even though they may have expressed an interest in receiving THN. Targeting prisoners who were due to be released was simpler for some training prisons with longer sentence prisoners, whilst remand prisons struggled with the prisoner ‘churn’ (the throughput of prisoners within a prison); thus, the process used to identify prisoners on arrival ‘did not catch-up’ with short-term or remand prisoners who arrived at an establishment and left within a short period of time. The process of identification and engagement of suitable prisoners and delivery of training to them was best suited to prisoners residing in a particular prison for some time. Short-term or remand prisoners, an important cohort of prisoners that may require the training, often did not receive it because of the speed at which they moved across the criminal justice system. Prisoners and staff also highlighted the need to ensure the availability of THN at other key points on a prisoner’s journey after being released. A number of prisoners who were engaged by staff identified the high risk of possible overdose within hostels or half-way houses immediately after release and also the need for an enhanced support and access to naloxone at this point.

The training [is] good and I’m glad I have done it, a real eye opener a lot of stuff I didn’t know. I know a lot of the lads think that ‘cos they [are not] using in jail they don’t think they need it but that’s not the case on the out [outside prison]. Best to get them…when they hit the hostels and it’s [drugs] around them again when they might be tempted [to use]. [Male prisoner, Prison 2]

### Realities of distributing THN within a prison setting

The staff highlighted issues with respect to the process of delivering training and the distribution of kits to prisoners at the point of release. At the time of the study, only clinicians were able to deliver the THN training as part of Patient Group Directions (PGDs), often within Immediate Life Support (ILS) training, thereby limiting the possible avenues by which THN could be introduced to prisoners. Peer mentors were identified as a potential group who could support the delivery of THN in prisons, but this process was just starting during the fieldwork (in April 2015). Moreover, there were important process issues underpinning the delivery of the actual kits. All ten prisons highlighted slightly differing approaches to placing THN kits within a prisoner’s personal property, including the use of ‘props’ (property) or ‘vals’ (valuables). Although there were broadly consistent procedures, the way an individual’s personal property was held varied slightly in different establishments. Consequently, the THN kits were often held at different points and places in the prison system, leading to difficulties in ensuring that the THN kits were distributed correctly at the point of release. Unlike other system-wide community-based settings such as Massachusetts (15), the distribution of the kits was also perceived to be under the remit of healthcare as ‘medication’, and only prescribers or staff with the requisite PGDs could supply the kits directly to prisoners. Clinical staff also expressed a sense of ‘fear and apprehension’ in allowing the distribution of THN kits to non-clinical teams. For some, clinical oversight was necessary to ensure the safe distribution of THN to prisoners as part of a wider package of support.

I am very concerned that Naloxone could be given out by prison officers or anyone else without healthcare input. The risks will be too great if something went wrong and the wrong person got hold of it. [Healthcare nurse, Naloxone Action Group]

As a consequence, the majority of the training delivered during the lifetime of the fieldwork was placed within a basic ILS skills training context, which included the wider relapse prevention and overdose work. There was also a lack of clarity over whether a THN kit, including an intramuscular needle, should be allowed within a court setting (for prisoners on remand) and whether it should be placed within their personal possessions. It remained unclear what would happen to a THN kit in this scenario, with a suggestion that possession of a kit would be automatically confiscated. Other procedural problems included contractual difficulties with some providers. In some prisons, the introduction of THN had not been embedded within existing contracts, and thus, there was a concern over resourcing the THN distribution. There was the possibility that providing this service would lead to problems accruing elsewhere within the service, balancing the economic costs of choosing this activity over another competing demand.

### Engagement of senior prison staff is essential

The qualitative interviews also included a strong message that the cooperation and proactive support of the prison governor was essential in ensuring progress in the implementation of the programme. A wider ‘whole systems’ approach was advocated, whereby implementation was placed within the remit of the prison’s Drug Strategy to ensure the most effective distribution and to avoid an unnecessary burden from being placed on the healthcare teams. For example, one prison highlighted that over 40 staff members, including community representatives, had been trained in delivering THN. The aim of this approach was to create a support mechanism by which other prison staff could help with training or managing processes to ensure the effective distribution of kits.

Participants believed that engaging the whole prison system would result in a degree of ‘culture change’, whereby all the staff could perceive the benefits of THN. Participants also highlighted the problem-solving nature of most prisons. Problems could be solved quickly if they were identified by the senior management, and there was sufficient ‘buy-in’ from operational staff. The need to ensure that the senior prison management are engaged in the programme was considered to be essential.

## Conclusions

The findings from the qualitative interviews identified a range of barriers to the implementation of THN across ten prisons in one English region. A number of the themes identified in this study were consistent with the wider community-focused literature. Despite these similarities, stakeholders suggested that distribution of THN kits in prison is a task fraught with difficulties that requires a ‘whole system’ approach. There is a need to simultaneously tackle negative staff perceptions and develop clear processes to ensure eligible prisoners are trained and given access to the THN kits. The key finding from this study is that it is insufficient for prisons to merely offer training and distribute kits to opiate-using prisoners without conducting a more enhanced planning and preparation process. To achieve this effectively and to minimise levels of disengagement, two main elements will need to be addressed.

Firstly, any prison-based training programme needs to reduce loss of potential trainees through the complexity of the prison system. This requires a detailed mapping of prison processes and procedures, where prison staff establish local processes to identify eligible prisoners (e.g. opiate users) and intervene at the most effective point in the prison journey. Prison leadership is important in ensuring that such processes run effectively, and any concerns expressed by prison staff are immediately addressed. This study has suggested that there are mixed views with respect to the appropriateness of distributing THN amongst both healthcare staff and prison officers, and the process of distributing THN kits is different across prisons. Relying on healthcare teams to be the sole route for THN delivery may miss other more efficient ways to ensure the kits’ distribution to prisoners at the point of release, including the prison resettlement teams. This is mainly a function of the prescribing described above, and at the time of writing, there were plans to allow non-clinical staff to distribute THN kits directly to prisoners.

The problems highlighted in identifying and tracking opiate users within the prison resulted in key groups (e.g. remand or short-sentenced prisoners) often being excluded from the THN distribution process. This is a major limitation in the design of an effective training and distribution programme. An alternative option would be to enhance the coverage of training to all prisoners regardless of sentence length or time within the criminal justice system. Given the role of bystanders in providing ILS and supporting possible overdose situations, there is an opportunity to widen access to THN training to all prisoners. This has a number of key advantages. Firstly, widening coverage will ensure that no prisoners will be missed from training including those who may be affected by the stigma of being identified as a drug user. Secondly, it offers the opportunity to address the tension between providing harm reduction services within an increasingly abstinence-driven policy environment. Finally, there is a wider goal of training prisoners in life-saving techniques that could be used in other non-overdose-related situations.

The second key finding from this study is the need to incorporate a more nuanced consideration of the beliefs and perceptions of prisoners to ensure the effective distribution of THN within a prison setting. Training programmes have to provide the basics of life support and THN injection administration but also need to address fundamental fears about use of THN in the community. Prisoners expressed concern over the perceived side effects of THN, largely based on word-of-mouth discussions with other prisoners. Furthermore, there was the challenge of providing a harm reduction initiative in a largely abstinent and recovery-focused environment. Gaston et al. [12] have highlighted the role stigma plays amongst service users in recovery in the community, and prisoners described a mixed message that emphasised both the desirability for complete abstinence at the point of release and an acceptance of potential involvement in drug use at some point post-release from prison.

Consideration should also be given to the time at which the THN training and the wider offer is made to prisoners. Training at the initial point of entry into prison was seen as largely ineffective, because many prisoners were not at a point to consider naloxone as an intervention option so soon after arriving at an establishment. Hence, the staff suggested that it was better to wait until prisoners were ‘settled’ and more amenable to the harm reduction message. Furthermore, strategies to manage the process of THN distribution as prisoners move across the criminal justice system need to be developed. For prisoners on longer sentences, there seemed little viability in introducing THN at an early or mid-point in their sentence for the fear of any learning from the training being lost. Despite this, there is a need to ensure that prison staff provided a coherent message about THN throughout a prisoner’s time in the criminal justice system, as negative attitudes to naloxone may harden at other points prior to release. Staff suggested a focus on the attitudes and perceptions of opiate users whose sentences were coming to an end and stressed the need to offer a package of support that specifically addressed prisoner concerns about the use of THN. Finally, a consistent message should also be provided by community-based services, including raising awareness of THN amongst the probation service and local police forces.
